# An Account of the Efficacy of Mercury in the Cure of Inflammatory Diseases, and the Dysentery

**Published:** 1787

**Authors:** James Lind

**Affiliations:** Physician at Windsor, and Fellow of the Royal College of Physicians of Edinburgh


IV. *An Account of the Efficacy of Mercury in the Cure of inflammatory Diſeaſes, and the Dyſentery. Communicated in a Letter to Dr.* Simmons, *F. R. S. by* James Lind, *M.D. F.R.S. Phyſician at Windſor, and Fellow of the Royal College of Phyſicians of Edinburgh.*

IT is now pretty well known, that mercury is univerſally uſed, in the Eaſt Indies, as a

ſpecific in inflammations of the liver. This practice would to us, perhaps, appear empiri-cal, had not its ſalutary effects removed every doubt of the propriety of its uſe: it has alſo been employed with advantage in this country by ſome eminent phyſicians, in the cure of a few other caſes of inflammation; and lately, mercury, joined with ipecacoanha, has been adminiſtered in India with ſurpriſing ſucceſs in the cure of dyſenteries.

From theſe circumſtances I am led to believe, that mercury is poſſeſſed of antiphlogiſtic powers, that deſerve the attention of every medical practitioner. I have therefore been induced to collect the following inſtances of inflammatory diſeaſes in which mercury has been uſed with advantage; and I ſhall at the ſame time endeavour to point out the caſes of inflammation in which it may be hurtful.

I ſhall begin with the hepatitis, a diſeaſe which occurs ſo ſeldom in Europe, that its exiſtence has been doubted by ſome eminent phyſicians*, but which is very common in the Eaſt Indies†.

* Hofman. Opuſc. Patholog. Pract. Dec. 2, Diſſert. viii. Page 484.

† It is equally rare in the Weſt Indies as in Europe.

When the diſeaſe is *original,* and not induced by remitting fever, or other diſorders, it begins with a tenſe, heavy, but ſometimes pricking pain under the right hypochondrium, with a great increaſe of pain on preſſing the liver upwards, or when the patients lie on their left ſide. The eyes are generally tinged with yellow, and the patient has a ſharp pain on the top of the right ſhoulder, which is the pathognomonic ſymptom of this diſeaſe. The pulſe is ſometimes quick and ſtrong; at other times it differs little from the natural.

As the diſeaſe advances, the ſkin commonly becomes of a dark, ſallow colour; the reſpiration becomes difficult; and the patient is oppreſſed with ſickneſs, frequently accompanied with vomiting and purging of bile. If the inflammation continues, ſhiverings and ſuppuration ſpeedily enſue. When the matter points outwards, and is diſcharged by inciſion, the patient frequently recovers; but ſeldom, when the abſceſs burſts inwardly; death following either immediately or ſoon after, the patient becoming tabid, from the purulent matter that is diſcharged into the abdomen or thorax.

In the Eaſt Indies, as ſoon as they are convinced that the liver is affected, from its being painful painful when preſſed upwards, before the pain on the top of the right ſhoulder comes on, they take away a little blood, and, confining the patient to a proper antiphlogiſtic regimen, begin to rub in mercurial ointment on the ſide affected, and to give repeated doſes of calomel internally, loſing no time in bringing the mercury to the mouth; for as ſoon as this is effected, the pain commonly ceaſes, and by the time the effects of the mercury are over, which generally happens in a fortnight or three weeks, the patient's recovery is complete.

On the ſecond or third day of the treatment, they ceaſe to rub in the mercurial ointment on the affected ſide, but apply a bliſter on it, and the ointment is then rubbed on the other ſide.

When the diſeaſe has been neglected in the beginning, or the patient has had frequent returns of it, it is not eaſily conquered, but becomes chronic, and laſts for months, or even years. The liver being now diſeaſed, there is commonly a want of bile, and the patient is extremely coſtive. Mercury alone is inſufficient for the cure; but a change of climate becomes neceſſary, together with riding on horſeback, the uſe of gentle laxatives, ſmall doſes doſes of rhubarb mixed with fixed alkali, light food, ripe fruits, rennet whey of goat or cow's milk, &c.

Although the univerſal practice in the Eaſt Indies, of curing hepatitis by mercury, ſufficiently proves its power of checking inflammation, yet it is at times attended with ſeveral inconveniencies; ſuch as, bringing on a violent mercurial diarrhœa, by the medicine being thrown in haſtily, which muſt always be done, otherwiſe, in this diſeaſe, ſuppuration would ſoon come on; and in ſome caſes the ſalivation runs ſo high as to be truly troubleſome. In one inſtance of chronic hepatitis, I ſaw a mercurial hæmoptoe brought on by a long uſe of mercury; and conſtantly the patients are ſo much debilitated by taking mercury, that it is a conſiderable time before they perfectly recover their former ſtrength in a warm climate.

When the hepatitis has been induced by a remittent fever, or diſeaſes accompanied with putreſcency, the uſe of mercury is always attended with the worſt conſequences; which ſeems to ſhow, that its antiphlogiſtic power muſt either depend on its inducing the putrid diatheſis, or that the atony which mercury brings on*, quickly removes the inflammatory ſpaſm. Thus the ſtrength of the ſtrongeſt man is ſoon reduced, when he is put under a courſe of mercury for a trifling venereal chancre, which, were it left to itſelf, would not impair his ſtrength in many months: but from which ever of the two cauſes the antiphlogiſtic power of mercury may depend, practice ſhews that it ought never to be uſed where there is putridity.

Doctor Gilchriſt, in the third volume of the Edinburgh phyſical and literary Eſſays, gives us ſeveral convincing proofs of its utility in this country, in a diſorder frequently attended with inflammation, which he terms a thickening of the urinary bladder, a diſeaſe which, he ſays, is ſometimes cauſed by, and frequently is attended with inflammation. In his firſt caſe, of a woman who had a round tumor, which was felt riſing two or three inches above the os pubis internally, after bleeding and laxative fomentations, liniments and proper drinks had been uſed, the mercurial pill was given, by which the illneſs was preſently removed. He

* It is probably from the atony it induces, that mercury has been found ſo ſerviceable in the tetanus.

obſerves, obferves, this was perhaps no more than a fimple inflammation of the bladder *.

In another cafe which he relates, of a gentleman near fixty, " a fmall bleeding," he tells us, " fhewed the blood a little fizey, and feem-" ed to moderate fymptoms; but a fecond, " ftill fmaller, gave neither the fame appear-" ance in the blood, nor the fame relief, and " his pulfe flagged. The tumour and inflam-" mation were great; there was no room for " evacuations, nor was any time to be loft. " The only refource left, that could be depend-" ed on, was the mercurial pill; by a few " dofes of which a fenfible abatement of pain " was procured †."

Speaking of this method of cure, and ufe of the mercurial pill, " Many," fays he, " would have queftioned the propriety of a " mercurial remedy; and I fhould have doubt-" ed of it likewife, if experience, in cafes of " no lefs delicacy, had not long before con-" vinced me both of its fafety and utility. A " lefs-powerful deobftruent did not appear " adequate to produce thofe great and fpeedy

* Effays and Obfervations Phyfical and Literary, Vol. III. page 474.

† Ibid. page 478.

 effects

"effects which the vehemency of the distem-

"per required. Besides I made choice of the

"most simple and harmless preparation of

"mercury *."

And in another place †, speaking of its effects, he observes, that "even before it is felt "in the mouth it may have a considerable effect; but when that happens, a revulsion is "plainly made, and resolution is begun; in "consequence of which, the inflammation and "tenderness of the tumour gradually abate:" and he adds ‡, that "quicksilver is a powerful "antiphlogistic, and removes inflammation "without accelerating the motion of the fluids, "which it rather diminishes by subduing their "inflammatory disposition.

"When there is little or no fever," he adds, "it as powerfully resolves obstruction, "without diminishing the impetus of the "blood, on a proper degree of which resolution depends."

In several cases of inflammation of the bowels which have fallen under my knowledge,

* Essays and Observations Physical and Literary, Vol. III. page 493.

† Ibid. page 496.

‡ Ibid. page 498.

repeated repeated doſes of calomel, given till the mouth was affected, removed the inflammation after the ordinary methods had proved ineffectual. Crude quickſilver, for the moſt part, muſt be injurious, unleſs we ſuppoſe the diſorder to proceed from introſuſception, which it may remove mechanically; for its effects in common caſes, acting as an extraneous, ponderous body, muſt always be hurtful, unleſs ſome of it, being divided by oily or mucilaginous matters in the ſtomach or inteſtines, and thus entering the ſyſtem as a mercurial medicine, were to remove the inflammatory ſpaſm; but this it never will effect with the ſame certainty or ſafety as a ſmall quantity of quickſilver made active by being divided with the bland mucilage of gum arabic: therefore the practice of giving crude quickſilver is now, properly, almoſt laid aſide.

To give mercury in pleuriſy or peripneumony, is a practice that the moſt daring empiric in this country would ſtagger at; † but there are many inſtances of its being uſed with advantage in thoſe complaints in an hoſpital at Naples.

In ſeveral inſtances of inflammation of the eyes, where no venereal taint could be apprehended, I have found calomel, given over night before a cooling purge was adminiftered, carry off the inflammation, when the purge without it was never attended with any good effect; and, in fact, mercury is fet down as a remedy for ophthalmia in the Edinburgh Pharmacopœia Pauperum.

The ufe of mercury, in curing inflammations attending ulcers on the legs from broken fhins, is known to moft furgeons, and therefore it needs not now be taken notice of; as is its power of removing every inflammation arifing from a venereal caufe.

Pimples, when attended with any degree of inflammation, are fooner cured by rubbing a little mercurial ointment on them than by any other application I am acquainted with.

Dr. Clark, in his excellent book, entitled, Obfervations on the Difeafes in long Voyages to hot Countries, and particularly on thofe which prevail in the Eaft Indies, gives an account of mercury's never failing to cure expeditioufly fixed pains in chronic rheumatifm, when it has been confined to fome particular part of the body, as the fhoulders, the joints of the knees, and the arm, after it has refifted every ufual remedy; a practice which he learned from from Dr. Silvefter. At firft, the great fuccefs that attended this practice induced him to believe the complaint was joined with venereal pains; but he afterwards found it equally efficacious in other cafes, where there was no reafon to fufpect any venereal taint.

One of the moft ufeful purpofes for which mercury has been given is, that of curing dyfenteries—a practice which has been lately followed with the greateft fuccefs on the Coromandel coaft. It was firft made known to the different furgeons in the Carnatic by a letter fent to each of them from the late Mr. Paifly, firft furgeon of the Prefidency of Madras.

Their method is as follows:—As foon as the patient begins to complain of fymptoms of dyfentery, they give him repeatedly fmall dofes of emetic tartar till it operates upwards and downwards, and thoroughly clears the ftomach and bowels; after which they begin to give mercury combined with ipecacuanha in the following form:

℞. Argent. viv. ℈j.

Pulv. gum arabic ℈ij.

Aq. puræ q. f.

Tere in mortar. marmor. ad perfect. extinct. globulorum, et adde

Pulv.

Pulv. rad. ipecacuan. ʒj.

Fiat maſſa dividenda in pilulas clx., quarum capiat unam, tertiâ vel quartâ quâque horâ.

This medicine they uſe till the urine, which in the beginning is high coloured, becomes pale, which they look upon as a ſign of the diſeaſe being ſubdued; after which a few opiates and ſome ſmall doſes of rhubarb, mixed with abſorbent powders, generally complete the cure.

During the courſe of the diſeaſe, they do not neglect to adminiſter emollient and ſtarch clyſters; and on the Malabar coaſt, where they had not yet* got into the practice of uſing mercury in the cure of dyſenterics, if the patient had much griping, they put a bliſter upon the belly, which, they were of opinion, likewiſe prevents inflammation and mortification, the ſymptoms moſt to be apprehended in this diſorder.

It is probably from mercury preventing inflammation, and conſequently mortification, that the above practice is ſucceſsful. Mr. Wilſon, an ingenious ſurgeon in the ſervice of the Hon. Eaſt-India Company, told me, when at

* In 1780.

Pon-

Pondicherry, that he had feldom loft above two men in a year by dyfenteries in the battalion of feapoys to which he was furgeon, fince he became acquainted with the practice of ufing mercury in this complaint; whereas before that he frequently loft in the battalion from twenty to thirty men by dyfenteries in a fickly feafon.

It is to be obferved, that the diforders on the Coromandel coaft are attended with much lefs putridity than thofe of Bengal, and mercury ought therefore to be ufed with great caution in Bengal, or wherever there are figns of putrefaction. From this circumftance it is that we may expect the greateft advantage from mercury in the cure of dyfenteries in Europe, where the inflammatory diathefis is moft prevalent. Indeed the practice is not altogether new to us; for the ufe of calomel mixed with rhubarb is ftrongly recommended by Sir John Pringle in his Obfervations on the Difeafes of the Army, and its utility is fully confirmed by the practice of many who have ufed it.

Wherever I have adminiftered mercury in the cure of inflammations, I have always ufed the two following preparations of it as the leaft ftimulating, viz. for external ufe, mercurial ointment, ointment, made with hog's lard alone, or with the addition of a little bee's wax to the lard first employed in killing the quickfilver, when in a hot climate, where the lard, from being too fluid, has not tenacity enough to divide the quickfilver; and, for internal purpofes, I have always divided it with mucilage of gum arabic, which is an efficacious as well as eafy manner of preparing mercury.

In the cure of inflammatory diforders by means of mercury, we ought carefully to guard againft inducing any great degree of falivation, by which many dreadful fymptoms are brought on; and as the fubject is new in this part of the world, practitioners ought to be circumfpect and cautious in the adminiftration of it; by which means, I hope, fair and candid experiments, not prejudice, will determine whether the practice is ufeful or injurious.

*Windfor,*

January 3, 1787.

